# Urban residents’ self-rescue in response to public health emergencies in China: a qualitative study

**DOI:** 10.1186/s12889-023-17442-5

**Published:** 2023-12-16

**Authors:** Yazhuo Gao, Ying Chen, Yin Lin, Fangfang Zhong, Xuehua Zhu

**Affiliations:** School of Nursing, Zhejiang Chinese Medical University, Hangzhou, 310053 China

**Keywords:** Public health emergency, Urban residents, Emergency self-rescue ability, Qualitative study

## Abstract

**Background:**

The abject uncertainty and unpredictability of public health emergencies have plagued various countries. Global health governance and international communities are facing long-term and arduous challenges. The self-rescue ability of individuals in a public emergency may be the most powerful trait to improve the survival rate outside the hospital. The study explores the cognitive ability and attitudes of urban residents in China towards self-rescue in response to public health emergencies. It provides appropriate evidence for improving the self-rescue ability of urban residents in China.

**Methods:**

Sixteen urban residents were selected using the purposive sampling method for semi-structured interviews. Theme analysis was used to collate and analyse the interview data.

**Results:**

Two themes and five sub-themes were analysed. The two themes included cognition and attitude of Chinese urban residents for self-rescue in an emergency. Urban residents believed that their knowledge and skills for self-rescue in an emergency were low. The ability for emergency self-rescue is affected by multiple factors, with relatively limited options for improvement. Nonetheless, the respondents expressed a desire to accept interventions under psychological crisis and a strong willingness to acquire knowledge and skills required for emergency self-rescue.

**Conclusion:**

This study investigated the perceptions and attitudes of Chinese urban residents towards emergency self-rescue. The results support enhanced ability of urban residents to respond to public health emergencies, thereby diminishing the negative outcomes. The findings suggest the need for strategies to address the factors affecting emergency self-rescue.

Public health emergencies (PHEs) are currently defined as unforeseen events that seriously affect public health, causing serious damage to the health and well-being of population. PHEs include major infectious disease outbreaks, unexplained diseases, major food and occupational poisoning events, among others. Other events that seriously affect public health mainly involve biological, chemical, nuclear catastrophes, terrorist attacks, natural disasters, including floods, droughts, earthquakes, fires, and mudslides, as well as major environmental disasters.. Casualties and epidemics due to accidental events also affect public health [1].

Public health legislations, policies, and regulations in China have been formulated retroactively after major public health emergencies. However, after the outbreak of severe acute respiratory syndrome (SARS) in 2003, the Chinese government promulgated a series of laws and regulations and formulated a series of emergency management procedures to improve the quality and effectiveness of public health emergency legislation in China [2]. Public health emergencies may result in unprecedented mortality and morbidity directly and indirectly, which can overwhelm the capacity of local governments equipped to manage traditional disasters [3].

The COVID-19 pandemic caused by SARS- coronavirus (SARS-CoV-2) has led to rapid and significant challenges worldwide. It is an extreme event with low probability and high loss, and has seriously affected the population health, health care system, and socio-economic conditions around the world [4].

Although we are currently in the post-pandemic era, COVID-19 has exposed the lack of public health capacity and the tensions between national sovereignty and global cooperation in dealing with PHEs. In addition, when dealing with various types of PHEs, the response measures are often constrained by limited social resources, medical, economic, legal, and population benefits, thereby hindering optimal disease control [5]. Therefore, the abject uncertainty of the risk of PHEs continues to plague countries. Adequate preparation in response to these threats requires global health governance and overcoming the challenges facing the international community.

Studies have analysed individual ability for self-rescue under a new term "disaster literacy". Disaster literacy is defined as individual ability to understand, evaluate, and apply disaster information to arrive at the right decisions for implementation of disaster-related instructions in daily life, thereby maintaining or improving the quality of life. The concept is crucial for public and societal understanding of disasters and the ability to respond to disasters [6]. Studies suggest that public response is an important component of the PHE response mechanism [7–9]. The diverse roles played by the public, including on-site witnesses and victims, are closely related to the handling of PHEs. The ability of the public for self-rescue during emergencies to ensure personal safety and protection via early warning systems, self-rescue strategies, and mutual assistance, can be enhanced by appropriate planning, learning, and training interventions [10].

Nonetheless, knowledge, attitude, and practice contribute to self-rescue in PHEs [11]. Urban residents constitute the main component of social groups, and thus have a significant impact on the community, medical institutions, and even the government. Zhang and colleagues believe that changes in knowledge are easier and quicker than changes in attitude. However, the changes in individual behaviour are more difficult and time-consuming than those involving knowledge and attitude [12]. Therefore, the current levels of knowledge, attitude and practice among urban residents are of practical significance in PHEs. Existing data show that the emergency self-rescue ability of the resident population may be the most powerful trait contributing to enhanced survival in PHEs outside the hospital [13, 14]. Therefore, the education and training of urban residents in emergency self-rescue operations may lead to positive outcomes.

Studies have shown that developed countries worldwide strongly emphasize the importance of emergency self-rescue capabilities. The developed nations have invested substantial manpower, material and financial resources to emergency self-rescue interventions. In Norway, 89% of middle-school students receive basic life support training [15]. Relevant laws and regulations required every resident to undergo emergency training in Switzerland [16]. Nearly 70% of the population in the Republic of Slovenia attend courses in cardiopulmonary resuscitation [17]. The rate of emergency training in Australia is also as high as 60% [18]. However, the emergency training rate in China is less than 26% [19]. Therefore, understanding the cognitive abilities and attitudes of Chinese urban residents for emergency self-rescue in response to PHEs is crucial to promote behavioural changes among individuals across various regions of China.

Invariably, a few developed countries have acquired substantial experience in improving emergency self-rescue ability. International studies focus on training, evaluation, and implementation of emergency self-rescue capabilities via regular simulation exercises and comprehensive and diverse measures for social emergency preparedness. However, the promotion of emergency self-rescue capabilities in China is still in its infancy. Few studies have reported the cognition, experience, and attitude of residents in response to PHEs. Therefore, the purpose of this study is to conduct semi-structured interviews involving Chinese urban residents to investigate their cognition and attitude towards emergency self-rescue, in order to improve and enhance the application of emergency self-rescue strategies.

## Material and methods

### Research methods

This study used purposive sampling to recruit respondents from communities, businesses and schools in Hangzhou, Zhejiang Province. The number of respondents was determined after comprehensive evaluation based on the principles of qualitative research analysis by eliminating duplicates and ensuring that no new topics were found [20]. Data from 14 interviews reached saturation, which was confirmed by two additional interviews. The informed consent of participating urban residents was obtained, and no participant withdrew from the study. Finally, a total of 16 respondents agreed to participate in the study. The participant demographic information is shown in Table 1.

**Table 1:** Demographic characteristics of the respondents (*n* = 16)

Variable	Frequency	%
Gender		
Female	5	31.2
Male	11	68.8
Age (years; mean and range)	37(19–58)	
Occupation		
Student	2	12.5
Teacher	3	18.7
Government employment	3	18.7
Private sector	5	31.3
Individual business	3	18.7
Highest educational level		
Master 's degree	4	25.0
Bachelor 's degree	5	31.3
Specialist qualifications	2	12.5
High school and below	5	31.3
Had received first aid-related training		
Yes	10	62.5
No	6	37.5
Ethnicity		
Chinese	16	100

### Interview outline

An interview outline was developed based on previous literature reviews [21, 22] and expert consultation. The interview questions were as follows: (1) Do you know what 'public health emergency' is? What do you know about PHEs? (2) What do you think of 'self-rescue'? How do you define the concept of 'self-rescue'? (3) If you encounter all kinds of PHEs, including major infectious diseases, food poisoning, and chemical leakage, how well can you save yourself? (4) What self-rescue ability do you think the public needs to have in various types of PHE? Can you provide specific examples to illustrate specific behaviors reflecting these abilities? (5) What factors do you think will affect your self-rescue ability? How do you think you should improve your self-rescue ability? (6) Can you give some advice or suggestions to improve the self-rescue ability of urban residents to deal with PHEs?

### Data collection methods

The interview was conducted in a relatively quiet location, without visual or audio interference. The interview lasted for 30 to 50 min (mean ± SD: 42.5). Before the formal interview, the researcher briefly introduced the study purpose, content, and significance to the interviewees. Further, complete confidentiality was promised, including replacing the name with the number A-P. The participants’ informed consent was obtained. The researchers recorded the interview site, observed the expressions and body movements of the interviewees, and recorded the whole process.

### Data analysis

Data collection and analysis were carried out simultaneously. The results of previous interviews were used to guide the direction of follow-up interviews. Thematic analysis entailed reading and analysis of the transcripts using a similar approach. The data were first grouped into codes using common keywords and quotes that were obtained from the interview transcripts, which described the participants’ perceptions and attitudes. Next, themes and sub-themes were developed. The participants’ demographic data were analysed using descriptive statistics.

### Quality control

The interviewer received systematic training in qualitative research, with proficiency in interviewing skills. The interviewer was supervised by an associate professor proficient in qualitative research methods. First, the transcription of the recorded interview was conducted promptly based on a standard protocol. Redundant and overly colloquial or non-substantial sentences were deleted. The electronic version was transcribed within 24 h after the interview, and was repeatedly verified by two or more researchers. Second, the interviewees were contacted to validate the interview content. The actual information was verified to ensure the authenticity, integrity, and accuracy of the data. If necessary, experts in related fields were contacted. Any conflicting opinions, deficiencies, and limitations were resolved by the researchers who contacted the experts promptly. Researchers promptly analysed each interview, decreased their influence on the results, and adjusted and supplemented the outline.

### Ethics approval and consent to participate

The study was approved by the Ethical Review Board (ERB) of the Zhejiang Chinese Medical University (DSRB-Ref 20,200,529–1). All participants were fully informed about how their data and information would be used in this study, and their right to withdraw their participation at any time. All participants were provided with an information sheet and the purposes of the study were described in-depth. All participants provided verbal and written consent to participate.

## Results

The study respondents included 11 males and 5 females with an average age of 37 years (range, 19–58 years). They included students, teachers, government and private sector employees, and self-employed individuals. Ten of the respondents received first aid-related training. Based on the qualitative data, two themes and five sub-themes were developed.

### Theme 1: cognition of emergency self-rescue ability by Chinese urban residents

#### Low levels of emergency self-rescue knowledge and skills

Currently, few studies investigate public intervention in response to PHEs in China, which seriously limits the ability of public response. Basic knowledge of PHEs is the most powerful tool in any response to emergencies, and represents a strong social foundation [13]. However, most respondents were unable to specify the types of PHEs, and were ignorant about the response to various types of events. However, the participants indicated that prompt treatment and management of emergency events such as food poisoning is a race against time. This finding is consistent with Lin Fei's results [23]. Notably, the researchers asked questions about the interview outline such as "what do you think of your own emergency self-rescue ability level?" All respondents agreed that their actual emergency self-rescue skills and knowledge of first aid were far from adequate. Nine respondents who were trained in emergency rescue were afraid to take action in PHEs due to a lack of professional competence and confidence, and inadequate ability for emergency self-rescue. It was impossible for most respondents to summarise the different types of PHEs, and few understood the response measures in various events.

*Respondent B: "I think the occurrence of major food poisoning and natural disasters are PHEs. If I suffer from food poisoning, I will seek medical treatment immediately. However, the treatment of other situations is not clear (shakes head)."*

Zhang and other experts believed that emergency experience and first-aid learning had a significant and positive relationship. Meanwhile, experience with past emergencies had a powerful impact on the recognition and evaluation of future emergency events [24]. Fortunately, some residents who have experienced PHEs realised their lack of emergency self-rescue knowledge and self-rescue skills.

*Respondent G: "In particular, the outbreak of the COVID-19 epidemic has made me realise that it is very important to master the knowledge and skills of self-rescue. We must always make personal preparations for possible PHEs."*

#### Emergency self-rescue ability is affected by multiple factors

In order to understand PHEs, many investigators only conducted simple, cross-sectional, epidemiological studies to determine objective influencing factors. For the public, the subjective factors of trauma and individual behavioural responses were largely ignored [25]. Therefore, in response to questions regarding self-rescue strategy during PHEs and the factors determining the ability for emergency self-rescue, most respondents believed that the self-rescue ability of urban residents was mainly affected by first aid experience, education levels, and the popularity of emergency self-rescue knowledge and skills. Individuals who have experienced PHEs or received relevant emergency rescue training have a significant advantage in self-rescue ability. Individuals who only acquired emergency self-rescue knowledge but lacked practical experience suggest inadequate combination of theory and practice. Only real experience can equip them with the knowledge, ability, and skills required for emergency self-rescue. Someone with skills and competence in first aid and emergencies can be of help to community, and can even save lives in any place and time.

*Respondent L: "I have received emergency rescue training, so I have a lot of experience. I think my *emergency* self-rescue ability is higher than that of people who have not received training."*

Education is also an indispensable factor. Residents expressed that individuals with a high level of education have more opportunities to absorb and acquire knowledge and skills from the outside world than those with a low level of education, and have stronger comprehension. This is consistent with the perspectives of some experts [6, 26]. Nonetheless, mastering the knowledge and skills of self-rescue in response to PHEs can improve the quality and ability of urban residents in emergency self-rescue, so it needs to be widely popularised.

*Respondent C: "I think highly educated people will have a higher desire for knowledge, and it is easier to obtain information and materials from different sources. But it would be better if it could be popularised."*

#### Limited ways to improve emergency self-rescue ability

In China, PHEs are primarily managed by national emergency medical rescue teams, civil rescue organisations, and medical institutions [27]. However, urban residents who are not affiliated with any organisation should be self-reliant during emergencies. Eleven respondents said that it was not easy to improve their emergency self-rescue ability. In the absence of personal initiative to acquire relevant information and identify learning resources, few opportunities are available for daily access to information related to PHEs. Notably, the need for improvement in emergency self-rescue ability of urban residents is unbalanced. Most of the residents said that emergency self-rescue knowledge can be primarily obtained through mobile phone or TV. A small number of individuals obtain such knowledge by reading newspapers and listening to radio. The overall results of the interviews suggest that mobile phones, computers, television broadcasts, and newspapers or magazines are not very helpful to urban residents.

*Respondent E: "The network environment is very free. I occasionally see relevant learning videos or *articles* on software applications, but I cannot learn systematically and it is difficult to remember completely."*

*Respondent P: "I usually use a mobile phone or computer to learn, mainly by watching videos. In *addition* to understanding some PHEs, I also saw the Chinese government's top-down propaganda. However, I think that the publicity of emergency self-rescue ability is far from enough."*

### Theme 2: attitudes of Chinese urban residents towards emergency self-rescue capacity

#### Desire to accept psychological crisis intervention

Studies have suggested that failure to address the mental health challenges of individuals recovering from crises such as natural disasters or PHEs may further aggravate the economic pressures in the country and lead to greater economic losses. Effective response measures are essential to cope with pain and interference, and thereby ensure the quality of mental health services [28]. Therefore, the researchers interviewed the participants based on mental health and emotional aspects. All respondents experienced the COVID-19 PHE. Thirteen respondents indicated that in the early days of the epidemic, in addition to serious effects on daily life and work, they also felt unprecedented psychological pressures, including feelings of shock, panic, and overwhelmed by the devastation. Studies show that psychological reactions triggered by PHEs included maladaptive behaviors, emotional distress, and defensive reactions. A specific syndrome called "headline stress disorder" was also detected, which was characterised by stress and anxiety caused by endless reports from the news media, resulting in adverse physiological symptoms, such as palpitations and insomnia [29]. This finding was consistent with the views of some respondents, who realised that they focused excessively on media information, without the ability to distinguish its authenticity. Daily emotions are closely related to news reports. Therefore, most of the respondents were eager to accept psychological crisis intervention to accurately manage PHEs, and thereby restore mental peace and normal daily life as soon as possible.

*Respondent A: "I think my emotions and opinions can easily become irrational because of the remarks of some online media. This should not become the norm. We should treat the problem dialectically with a rational attitude."*

*Respondent K: "The first thing I wake up every morning is to read relevant news reports. Because of the fear that my families may be infected by virus, sleep is also affected. I hope that in the future there will be professionals to carry out mental health education for the public.*

#### Strong willingness to learn emergency self-rescue knowledge and skills

PHEs such as the COVID-19 pandemic have led to mental and psychological challenges among Chinese urban residents. Residents found themselves working in a chaotic environment caused by disasters, which hindered return to normal lives [30]. Therefore, the researchers questioned the respondents and their willingness to improve their ability for emergency self-rescue. All respondents expressed high expectations for a comprehensive training in emergency self-rescue knowledge and skills. Twelve respondents reported that after realising their lack of emergency self-rescue knowledge and skills, the idea of spending time to acquire mental health-related content was very strong for adequate preparation for possible PHEs in the future. Nonetheless, some respondents suggested that the relevant emergency training should be organised and led by the government under a uniform training mode. A small number of residents also expressed the hope that emergency self-rescue training was free for public health.

*Respondent F: "I still prefer to take some time to get professional training in systems *because* these knowledge and skills are critical to saving lives. The training had better be free for the people. Public welfare emergency training is more attractive to us."*

*Respondent G: "I' m sure I' d love to participate in this training. I hope it's free, but if the training costs *money*, I might consider it."*

## Discussion

The results show that the urban residents participating in this study have a serious shortage of skills and abilities required for emergency self-rescue in PHEs. However, the residents' awareness of risk perception is low. A sudden disaster requires immediate response. In the absence of data to support such a response, residents are prone to panic, and fall into a passive state of simply waiting for government relief without taking any action. Yang et al. proposed the idea of establishing an emergency intelligence capability system [31]. In the era of digital social governance, the Chinese government and all sectors of society attach great importance to the construction of an emergency intelligence capability evaluation system in order to improve the rescue efficiency in major PHEs. The operational efficiency of the system determines the efficiency of emergency rescue and management, which not only facilitate emergency decision-making for rapid response in major public health events, but also provides important decision support for governmental decision-making. It is undeniable that in the past decade, cities in China have accelerated the pace of establishing relevant regulations required for emergency interventions and building emergency intelligence capability systems to avoid unnecessary casualties. These are important to improve the ability of urban residents to respond to PHEs [32]. However, urban residents have yet to acquire appropriate education and training skills for emergency self-rescue. Training is an indispensable component for self-rescue in PHEs. However, the survey shows that the quantity and quality of training do not meet the required standards [33, 34]. Timely self-rescue in an emergency can undoubtedly save lives in a timely manner. However, studies suggest that emergency self-rescue knowledge and skills deteriorate rapidly after initial training due to their loss over time [35]. Therefore, single or multiple sessions of emergency training are not enough to meet the needs of urban residents. Various strategies are needed to retain emergency self-rescue skills for a long time. For example, with the city government as the main force, administrative health departments and emergency agencies as auxiliary forces, emergency exercises can be designed to help residents practice and consolidate their knowledge and skills of emergency self-rescue under simulated scenarios.

Emergency self-rescue ability is an important aspect of personal first aid [36]. Effective prevention and response strategies are urgently needed in order to reduce the harmful effects of PHEs,. Even though some respondents recognised the importance of emergency self-rescue, they still believed that the ability is often affected by multiple factors and limited ways for its improvement. First, Mou believed that the low ability for self-rescue under PHEs is related to the lack of public participation and inadequate emergency preparedness [37]. The aforementioned findings and results of interview suggest that the public should improve their participation, raise awareness of risk perception, and strengthen emergency preparedness and foundation. The focus of public attention should be on emergency needs. The opinions and suggestions of members of the public as well as their experience should be encouraged. The emphasis should be on publicity and education, and post-training evaluation. The public should closely link their future with the future of the country. In the face of PHEs, the public should respond immediately. Second, the "National Comprehensive Disaster Prevention and Mitigation Plan (2011–2015)" issued by the State Council of China emphasises the principle of "prevention first, comprehensive disaster reduction intervention." According to the Plan, emergency education is indispensable to raise public awareness for prevention and response to PHEs via appropriate public education and training in disaster medicine and public health preparations. It can be seen that the government should actively implement educational programmes in emergency intervention and evaluate the characteristics, needs, and knowledge levels of the target population. According to the survey results, different forms of emergency education and training are needed for different groups of people to improve the risk perception and emergency self-rescue ability. Emergency education training and exercises should be led by the government to ensure universal, inclusive, and public participation. In recent years, guiding, coordinating, preventing and controlling the health emergency system has become the primary task of the Chinese government. In brief, the public and the government not only play an independent social role, but complement each other in fulfilling their social responsibility. Thus, the public's self-rescue ability can be enhanced in response to PHEs.

PHEs are a major test of the national governance system and capacity. China's organisational structure is based on the principles of delegation of responsibility and regional management at multiple levels of public health or medical emergency involving provinces, cities, and counties [38]. Wang et al. proposed two policy recommendations based on China's emergency response capacity, organisational structure, and levels of governmental management [39]. First, the whole process at each stage of emergency management should be balanced. The strategy is designed to address the shortcomings of China's emergency response capacity, which is "mainly based on disposal and light prevention," adherence to prevention and emergency preparedness, and preventing and resolving major risks and crises at the source. Second, the emergency coordination and regional coordinated development should be strengthened by enhancing the communication and interaction between provinces and cities, implement different developmental strategies according to local conditions, and accurately implement policies to overcome the shortcomings of emergency response capabilities. This study believes that the above suggestions provide a policy basis and a reference to build China's emergency capacity. They provide a direction for improving relevant policies in the future. Notably, the community is at the intersection of the government's emergency management function and social system. The unique nature of the community requires that efficient emergency management of PHEs by the government should be based on the dual role of system and individual [40]. Only by establishing the system or policy that enables urban residents to participate in emergency decision-making of PHEs can we effectively resolve social challenges and build a harmonious and stable living environment.

Studies have shown that the main laws and regulations used in the process of building a health emergency response system in China include 'Public Health Emergency Regulations', and 'People's Republic of China Emergency Law'. Nevertheless, few laws and regulations are related to public ability for emergency self-rescue in a crisis [41]. Therefore, the Chinese government attaches importance to changes in attitude towards the management of PHEs, in an effort to improve the laws, regulations and policies related to the emergency self-rescue ability at the population level [42].

PHEs represent a major threat to governments, decision makers and health care systems. Among them, major infectious disease outbreaks may create an unknown field due to a lack of understanding of the pathogen [43]. The spread of a viral disease may be very rapid, which can pose a serious threat to the health of residents [44]. SARS in 2003 and COVID-19 in 2020 seriously hampered the daily life and activities of public due to stringent prevention and control measures, and the suspension of all non-essential production and commercial activities hindered economic activity’ [45]. Whether or not it can be successfully controlled, a wide range of measures to control infection will inevitably have a psychological impact [46]. The study participant indicated that the psychological responses to the epidemic included emotional distress and defensive behaviours, such as anxiety, fear, depression, stress, and avoidance. Timely studies on the COVID-19 epidemic have confirmed the significant impact of the epidemic and psychological response measures, indicating high levels of psycho-pathological symptoms in the population [47]. Therefore, timely information release and accurate public opinion are particularly important at this time [48]. This is consistent with the views of Chen et al. [49]. According to Chen et al., social media contribute to crisis management during PHEs via real-time information sharing and interaction. The social media facilitate rapid self-assessment by stakeholders during a crisis. Government departments can also use social media as a tool to disseminate rescue and crisis information. However, media-based business and social platforms are concerned about the quality of information available. The massive information mixed with poor authenticity hinders public access and reading levels. Coupled with poor information dissemination, urban residents often focus on information and rumors. Therefore, distorted health information and rumors can adversely affect the mental health, judgment and behaviour of urban resident, leading to social panic. The government should actively control the news networks to under PHEs. The media should emphasise the facts to ensure psychological stability and rational decision-making by urban residents. Meanwhile, various information platforms should be strictly supervised and any harmful effects of false rumors on the public should be eliminated. Thus, we can effectively manage large-scale PHEs and respond to emergency needs under environmental uncertainty and information asymmetry during crises.

In addition, this study shows that most respondents expressed a desire to receive timely intervention in a psychological crisis. The findings reiterate the integration of public mental health interventions into plans for public health preparedness and emergency response [50]. A number of studies have reported the increased risk of psychiatric disorders such as depression and anxiety, and even post-traumatic stress disorder (PTSD) following PHEs [51–53]. However, other studies have found that adequate training can decrease the stress and psychiatric ailments [54, 55], for instance, during SARS. Nonetheless, support from family and society can also diminish the appearance of negative emotions [56].

Xiang and others suggested three important steps: institution of multidisciplinary mental health teams, clear communication with appropriate updates about public health emergencies, and establishment of secure psychological services and interventions [57]. The vulnerable populations must be specifically targeted in any psychological intervention, especially the elderly, the children, and the healthcare workers [58, 59]. Given the recommendation to minimise face-to-face interaction, online mental health services have been widely adopted in China with robust outcomes during the outbreak [60]. Positive psychological interventions can help people maintain psychological security and avoid self-harm caused by psychological imbalance. Therefore, the psychological crisis intervention must be a component of the public health response to PHEs. A new psychological crisis intervention model is needed. The ideal goal is to establish basic preventive procedures. Based on positive psychological intervention, urban residents can maintain the correct attitude to deal with any crisis and avoid a series of irrational behaviours. Therefore, it is necessary to implement psychological crisis interventions in PHEs.

## Study limitations

The limitations of this study are predominantly related to the number of Chinese urban residents who participated in the interview and the qualitative research methods utilised. The small sample size, limited by manpower and time, may weaken the representativeness of the selected samples. Further, the results may show relatively strong subjectivity due to the difficulties in conducting a quantitative analysis of the interview data using qualitative research methods. Studies in the future should recruit additional urban and rural residents across different occupations. Randomised controlled trials are desirable to obtain a wide range of perspectives.

## Conclusions

Urban residents represent an important category of the population in a society at risk of major public health crises. They should be responsible for their own health and well-being as well as social security. Therefore, it is critical to improve the emergency self-rescue ability of urban residents at the levels of population, community, and government. This study seeks to fill the gaps in our understanding and attitudes of Chinese urban residents towards PHEs. It explores strategies to overcome obstacles and improve the ability and quality of emergency self-rescue.

## Data Availability

The datasets generated and/or analysed during the current study are not publicly available but are available from the corresponding author upon reasonable request.
